# The relationship of prenatal maternal depression or anxiety to maternal caregiving behavior and infant behavior self-regulation during infant heel lance: an ethological time-based study of behavior

**DOI:** 10.1186/s12884-016-1050-5

**Published:** 2016-09-07

**Authors:** Fay F. Warnock, Kenneth D. Craig, Roger Bakeman, Thaila Castral, Jila Mirlashari

**Affiliations:** 1 Developmental Neurosciences, Child and Family Research Institute, L408, 4480 Oak Street, Vancouver, BC Canada; 2 University of British Columbia (BC), School of Nursing, Vancouver, BC Canada; 3 Department of Psychology, University of British Columbia (BC), Vancouver, BC Canada; 4 Georgia State University, Atlanta, Georgia 30303 USA; 5 University of Goiás Faculty of Nursing, Goiânia, GO Brazil; 6 School of Nursing and Midwifery, Tehran University of Medical Sciences, Tehran, Iran

**Keywords:** Prenatal depression, Prenatal anxiety, Maternal caregiving behavior, Infant pain, Infant pain behavior, Perinatal maternal mental health, Ethology

## Abstract

**Background:**

Sensitive and responsive maternal caregiving behavior strengthens infant self-regulatory capacities (HL), but this regulatory role may be diminished in some mothers with second-trimester prenatal exposure to depression and/ or anxiety (MDA). This study examined maternal and infant behavior during infant heel lance (HL) when mothers had or did not have MDA. Ethological methods and micro-analytic approaches capable of distinguishing and comparing time-based patterning in maternal and infant behavior were used to clarify biological mechanisms, such as MDA, that may underlie observed behavior. Aims were to examine group differences in caregiving behavior between mothers with and without MDA 5 min Pre-HL and 5 min Post-H, and relationships between MDA, maternal caregiving behavior and infant pain behavior self-regulation, concurrently.

**Methods:**

At second trimester, mothers were assessed for symptoms of mild-severe depression or anxiety. Mothers whose scores exceeded predetermined cut-off scores on one or more of the mental health measures were allocated to the MDA-exposure group, those below to the non-MDA-exposure group. Reliable observers, blinded to MDA status and study phases, coded video records of the caregiving behavior of each study mother for the full duration of the 5 min Pre-HL and 5 min Post-HL study phases. Group differences and associations between mean measures of maternal mental health scores, time-based measures of maternal behavior, and time-based measures of infant pain behavior regulation (previously coded) were concurrently analyzed using comparative and correlational statistics.

**Results:**

MDA-exposed mothers spent significantly more time not embracing, engaging or responding to infant cues than maternal controls Pre-HL and Post-HL. MDA was associated with atypical maternal caregiving behavior, which in turn was related to atypical infant pain behavior self-regulation during and after the HL.

**Conclusion:**

Our findings have implication for practice. We recommend inclusion of mothers with MDA and their infants in interventions that strengthen the early mother-infant interaction and mother’s regulatory caregiving role. MDA and maternal caregiving behavior must be considered in future infant pain studies to examine if they confound effectiveness of mother driven ﻿caregiving interventions ﻿for neonatal pain. We highlight the importance of examining maternal mental health throughout the perinatal and postnatal trajectory, and particularly the newborn period.

## Background

Sensitive and responsive caregiving actions and interactions (typically provided by the mother) play a crucial role in protecting, buffering and strengthening infant ability to self-regulate to everyday stressors [1]. This is important because, throughout the perinatal period, the self-regulatory capacities of the human infant continue to develop with rapid changes also occurring in brain structure and function. It is also during the neonatal period when most infants are exposed to painful clinical procedures such as heel lance (HL) for necessary blood screening and monitoring. Established evidence suggests that early exposure to everyday stressors including routine HL can overtax and disrupt the still immature newborn’s ability to self-regulate subsystems (autonomic, motor, state, attention, behavioral organization) [2], and confer damage to the structure and function of the developing pain system [3, 4]. This is particularly so if the infant is born at-risk (born with prenatal exposures, ill, developmentally compromised and/ or premature), and if the pain goes untreated [3, 4].

Currently, there are no analgesics that can be safely administered to infants to relieve procedural pain [5]. There is, however, substantial evidence that non-pharmacological pain treatments can effectively alleviate procedural pain in newborns based on a reduction in mean measures of pain behavior (primarily changes in facial movement), heart rate, and/or salivary cortisol levels [6, 7]. Examples include the use of oral sucrose as well as mother driven caregiving interventions (eg., kangaroo mother care, breastfeeding, facilitated tucking) [6, 7].

Growth in the number of maternal caregiving interventions for neonatal pain and their uptake into the clinical setting provides confidence in their reported effectiveness [6]. While in most of these interventions, the infant’s mother is acknowledged to play a fundamental role in regulating the infant and that the mother drives/functions as the “intervention”, there is limited reporting of the maternal sample beyond demographics (eg., age, socio-economic status). Under-reporting precludes understanding as to whether the interventions are representative of the diverse population of mothers and infants for which they are intended. Furthermore, lack in examining maternal factors prevents early recognition/detection of perinatal factors and mechanisms that could hamper a mother’s ability to regulate her infant, or that may function to confound effectiveness of the interventions. Findings of a recent narrative systematic review of 12 kangaroo maternal care intervention studies for neonatal pain [8], for example, show that none of the 12 studies provided information as to whether mothers with prenatal depression and/or anxiety were included in the sample. As well, none explicitly examined prenatal maternal depression and/or anxiety or maternal caregiving behavior as distinct study variables [8]. The consideration of these kinds of maternal factors in neonatal pain studies including mother driven interventions for neonatal pain is essential for the following reasons.

Depression and/or anxiety during pregnancy are considered some of the most serious and concerning issues confronting pregnant women and perinatal health practitioners [9]. Both often co-exist and confer increased risk for adverse maternal, pregnancy and infant outcomes [10]. Historically, the emphasis on prenatal assessment/screening, epidemiology, and research has focused on depression [10]. For example, prenatal depression has been shown to increase the risk for preterm birth, low birth weight, and intrauterine growth restriction [9, 11]. Recent findings, however, indicate that prenatal anxiety and stress are also associated with adverse maternal and infant outcomes and that anxiety related to the current pregnancy (pregnancy anxiety) may be especially potent [9]. Although exact mechanisms underlying pregnancy anxiety remain unclear, it is thought that potent infant effects may be due to complex interactions between maternal vulnerabilities that predate the pregnancy (eg., insecure attachment, lack of psychosocial resources) and anxieties a mother may have concerning certain aspects of the pregnancy [9]. These interactions are thought to increase levels of maternal anxiety that, in turn, influence the maternal-fetal-placental stress and hormonal systems in a manner that may contribute to functional changes in fetal hypothalamic–pituitary-adrenal (HPA) axis and to increased risk for adverse fetal neurodevelopment and premature birth [9].

Systematic review studies estimate that major depression, during pregnancy, affects up to 12.7 % of pregnant women [12] and that the prevalence rates (95 % CI) of depression for the 1st, 2nd, and 3rd trimesters of pregnancy are 7.4 % (2.2, 12.6), 12.8 % (10.7, 14.8), and 12.0 % (7.4, 16.7), respectively [13]. The preceding findings contribute to increased understanding of the prevalence of depression and anxiety during the trimesters of pregnancy. More recently, interest has shifted to also examining how the course of depression and anxiety throughout and at each trimester may adversely impact maternal, fetal and infant outcomes. Findings of a recent systematic review, for example, suggest that depression occurring during mid to late pregnancy may have the greatest adverse effects on fetal growth and development, while anxiety associated with preterm birth may contribute to changes in fetal HPA axis [14].

Studies that have linked maternal psychopathology with maternal caregiving have mostly involved mothers with postnatal depression. Findings show that some of these mothers exhibit a range of inconsistent or atypical caregiving behaviors such as being less or overly responsive to their infant cues, intrusive, or they withdraw from interacting with their infants [15]. Currently, it has been suggested that maternal caregiving behavior (eg., sensitivity) is a likely mechanism that links prenatal maternal depression [16] and /or anxiety [17] to infant and child bio-behavioral outcomes (e.g., difficult emotion regulation, altered cortisol patterns).

The previous findings highlight the concerning implications of 2nd-trimester exposure to maternal depression and/or anxiety (hereafter referred to as MDA) on maternal and infant outcomes as well as pointing to the potential mediating effect of maternal caregiving behavior. As it pertains to maternal caregiving interventions for neonatal pain, improved reporting and targeted recruitment of mothers with MDA and their infants into the study will help ensure the interventions are representative. However, more research is needed to clarify the significance of MDA during the neonatal period and if it underlies atypical maternal caregiving behavior as well as neonatal pain outcomes during routine pain procedures.

To our knowledge, there have only been two independent studies that have examined some of the linkages between MDA, and/or postnatal maternal caregiving behavior and/or newborn pain outcomes in full-term infants [18, 19]. Both were conducted by some of the authors of the current study (FW, KC, and RB). The first study [18] compared the proportion of time that mothers in three study groups [two groups of mothers with MDA (medicated with Selective Serotonin Re-uptake Inhibitor antidepressants (SSRIs) and not), and a control group] spent exhibiting atypical caregiving behavior as they held their infants while the infant had a routine HL. The findings showed that during the HL, both groups of mothers with MDA were more likely to be less responsive to their infant’s pain cry and to engage their infant less, compared to maternal controls. The second study [19] quantified the temporal profile of the behavioral responses of infants with and without 2nd-trimester MDA exposure during the sub-phases of the actual HL procedure (HL, post-HL). In that study, the HL was performed with the infant laying on a cot. Findings showed no group differences in the magnitude of initial behavioral reactions to the actual HL, but during the post-HL sub-phase, MDA-exposed infants spent more time crying in a weak/exhausted manner and in exhibiting strained and erratic limb movement and immobility. The findings indicated that infant prenatal exposure to MDA might have contributed to delayed recovery and diminished capacities for self-regulation of noxious distress in those infants.

Taken together, findings from the two studies [18, 19] indicate that MDA may underlie the observed atypical expression of maternal caregiving behavior and infant pain behavior. They further suggest that MDA and maternal caregiving behavior are significant factors to consider in neonatal pain studies and maternal caregiving interventions for neonatal pain. However, the first study [18] was limited in that findings were based on the brief event of infant HL (eg., 2 min). Hence, it is unclear whether mothers with MDA would exhibit similar patterns of behavior for longer periods of the HL session. The second study [19] examined linkages between MDA and infant pain behavior self-regulation, but not maternal caregiving behavior. To further clarify the significance and potential underlying impacts of MDA, it is necessary to examine the relationships of MDA to maternal caregiving behavior and infant behavior self-regulation concurrently and to examine maternal caregiving behavior over longer durations during HL.

This study addresses gaps and builds on findings of the two preceding studies [18, 19]. The study is part of a large project on MDA and its potential underlying effects on the caregiving behavior of mothers and newborn pain behavior self-regulation from which findings of infant pain self-regulation behavior reported by Warnock et al. [19] originate.

The first aim was to examine association and group differences in caregiving behavior between mothers with and without MDA before and following infant HL. To do this behavioral data were generated from the pre-recorded videotapes of the caregiving behavior of 24 mothers that had been collected but never coded or analyzed in the Warnock et al. [19] study. On the basis of findings of the first study [18], we hypothesized that MDA (as measured by maternal mental health scores taken at 2nd trimester) would be associated with maternal atypical caregiving behavior during a 5 min observation session before the infant was placed in a cot to have the HL (Pre-HL phase), and during a 5 min observation session after the infant had the HL and was reunited with their mother (Post-HL phase). We also hypothesized that during each of these two study phases, that the proportion of time that mothers spend exhibiting atypical behavior would differ between MDA-exposed mothers and maternal controls.

The second aim was to examine associations between maternal MDA-exposure, time-based measures of maternal caregiving behavior and the three atypical patterns of infant pain behavior self-regulation previously reported (crying in a weak/exhausted manner, strained and erratic limb movement and immobility) [19], concurrently. Data were time-based measures of maternal caregiving behavior generated from the 24 maternal pre-recorded videotapes as well as time-based measures of the three atypical patterns of infant pain behavior self-regulation. The infant measures were drawn from the pool of pre-calculated time-based measures of the 21 infants who had participated in the Warnock et al. [19] study. From the total samples of 24 mothers and 21 babies, there was a matched subsample of 16 maternal-infant dyads with complete time-based behavioral data to undertake the second study aim. Extending prior research [18, 19], we hypothesized that in this matched subsample of 21 mothers and their infants, maternal atypical caregiving behavior during the 5 min Pre-HL phase would be associated with the three atypical patterns of infant pain behavior self-regulation during the sub-phases of the actual infant HL (HL, post-HL).

## Methods

### Study design

This was an ethological micro-analytic descriptive comparative study that made use of systematic observation methods and time-based analytic approaches. We chose these methods and approaches because unlike conventional approaches, they are capable of generating precise measures of the proportion of time (Prop-T) that an individual or group spends in a particular behavior during an observation session(s), and that can be compared [20]. Furthermore, they have a unique ability in distinguishing patterning in behavior from the co-occurring running streams of behavior as the normally unfold, and in explicating biological mechanisms, such as MDA, that may underlie observed behavior [20].

Main outcomes of the current study were Prop-T time scores of maternal caregiving behavior and infant pain behavior self-regulation for the 5 min Pre-HL, 2 min infant HL (including post-HL sub-phase), and 5 min Post-HL study phases. Prop-T scores were calculated by dividing the full duration of time a mother or infant spent in a particular behavior by the entire duration of the respective study phase. MDA was operationally defined as mild to severe symptoms of prenatal depression and/or anxiety based on mean scores of prenatal mental health measures that mothers completed at the 2nd trimester. Descriptions of maternal recruitment and data collection procedures that we provide below are summarized from Warnock et al. [19]. This is because mothers of the current study were mothers of some of the infants from the Warnock et al. [19] study and because measures of MDA were collected from mothers prenatally. A further reason is that measures of maternal caregiving and infant pain behavior self-regulation were collected at the same time.

### Participants

As reported by Warnock et al. [19], mothers were recruited during the 2nd trimester of pregnancy (first visit) and then again on the day of their infant’s scheduled HL, which occurred about 36 h after infant birth (second visit). The 2nd trimester was chosen because of the increased prevalence and impacts of depression and anxiety compared to the 1st and 3rd trimesters, as noted earlier. Four mothers with MDA, who volunteered their participation were referred to the study by their primary care physicians who had diagnosed them as depressed and/or anxious. The remaining mothers were those who responded to advertisements of the study posted in health clinics, prenatal classes or newspapers. To be included, women had to be proficient in the English language, have no birth complications, no bipolar disorder or Axis II disorders, and their infants had to be >37 weeks gestational age with a birth weight of > 2500 g. Excluded were infants with congenital heart disease, central nervous system malformations and neonatal abstinence syndrome (NAS). Of the 36 mothers who initially volunteered, all met study inclusion criteria and all completed a mental health assessment at 2nd trimester of pregnancy. There were no missing data on any of the mental health measures that mothers completed.

Twelve of the 36 mothers were not included in the current analysis because of loss to follow-up during pregnancy (*n* = 1) or because of insufficient data on maternal caregiving behavior (*n* = 11). Of the remaining 24 mothers, six had reported taking drugs during pregnancy (SSRIs (*n* = 3), crack cocaine (*n* = 2) or SSRI + crack cocaine (*n* = 1). Those six mothers were not excluded from the current analysis in recognition that in our region, 5 % of pregnant women are prescribed SSRIs antidepressant medications [21] and 3.5 % report taking crack cocaine during pregnancy [22]. A further reason was that none of the infants had NAS based on pre-study screening by a neonatologist. However, because little has been published on the effects of prenatal drug use on maternal caregiving behavior during neonatal pain, “prenatal maternal drug use” (SSRIs and/ or crack cocaine) was entered as a covariate in the analysis as appropriate.

The final sample, therefore, consisted of 24 mothers. On the basis of a-priori sample size calculations of the previous study that involved infants of study mothers [19] (for Type 1 error rate of 0.05, a power of .80 and effect size of .30, ((G-Power 3.1)), a total sample of 24 mothers was needed to examine change and group differences in Prop-T scores of maternal behavior before and following infant HL. Based on Stevens [23], the sample size for each of the simple regressions involving one predictor variable required 15 subjects. The larger study from which this study and the Warnock et al. [19] study originate, received ethical approved from the University of British Columbia Research Ethics Board and by the Children’s and Women’s Health Centre Research Review Committee. All mothers gave written informed consent for both themselves and their infants.

### Procedures

#### Prenatal maternal mental health assessment

As reported by Warnock et al. [19], the prenatal assessment that the 24 study mothers completed at 2nd trimester and that took place during the first visit, included the well validated 10 item patient-rated Edinburgh Postnatal Depression Scale (EPDS), the well validated clinician-rated 21-item Hamilton Depression Scale (HAM-D) and the 14-item Hamilton Anxiety Scale (HAM-A). The three tools were administered to the mothers by a research assistant who was knowledgeable and trained in the tools, but not blinded to study aims. Mothers were assigned to the MDA-exposed group or the non-MDA-exposed group (control) on the basis of meeting predetermined cut-off scores on one or more of the mental health measures and that were capable of detecting symptoms of depression and/or anxiety or not. The cut-off scores were: HAM-D > 8 [24, 25], HAM-A > 8 [26], EPDS > 11 [27]. All three tools including same cut-off scores have been used in prior neonatal pain studies that involved mothers with MDA (medicated with SSRIs and not) [18, 19, 28–30].

#### Data collection: maternal and infant behavior

As per Warnock et al. [19], mothers and infants were assessed during the second visit in a quiet and heat regulated room in the hospital. Mothers were debriefed about the study procedures and advised that they could withdraw their participation at any time, or for any reason and to care for their infants as they normally would. Research staff did not interfere with the care that a mother chose to provide her infant during the HL procedure or with the routine hospital protocol for neonatal HL, which was performed with the infant laying on a cot. All HLs were performed by experienced lab technicians on infants who were awake and who did not have any HL in past 12 h. Also, data were based on a single HL.

Data collection commenced with mothers seated and holding their infants on their laps. One camera was positioned to capture full body views of the mother and the infant and to videotape continuously the behavioral responses a mother made to her infant for 5 min before the infant’s HL (Pre-HL). Upon arrival of the lab technician, the infant who was clothed in a vest and diaper was gently placed supine onto a cot by the infant’s mother or by the research assistant if the mother requested. A second camera captured body movements of the infant continuously for the duration of HL with crying simultaneously audio-recorded. All mothers remained in the room, and none chose to touch their infant. After the HL, the infant was reunited with their mother, and maternal behavioral responses to the infant were videotaped continuously for 5 min (Post-HL). The research assistant depressed a foot peddle to mark the beginning and end of each study phase and a running time was encoded on the study videotapes to enable second-by-second coding of maternal and infant behavior.

#### Maternal behavioral measures and coding procedures

In the current study, the pre-recorded videotaped cases of the 24 study mothers were each coded using the reliably established Maternal Behavior Coding System (MBCS) [18]. The MBCS is an ethologically based micro-analytic behavioral research coding tool that had been previously developed inductively for use with mothers with and without MDA during an infant pain event [18]. In a cross-validated sample, the MBCS distinguished typical from atypical caregiving behavior between two groups of mothers with MDA (medicated and not) and a control group of mothers [18]. The tool consists of main categories and subcategories of maternal caregiving behavior that are structured into two main domains–typical caregiving behavior and atypical caregiving behavior. We chose the MBCS because it enables trained coders to continuously code behavioral items second-by-second as the coders observe them to occur on pre-recorded videotapes from which precise Prop-T scores of behavior can be generated and compared (see Warnock et al. [18] for a copy of the MBCS for that study).

In this study, we drew on the coding procedures from the Warnock et al. [18] study to continuously code MBCS items from the 24 pre-recorded videotaped maternal cases. Two coders who were knowledge of the MBCS, trained in systematic coding, and who were blinded to maternal group and to study phase, coded the videotapes in random order, in terms of participants, using the MBCS. Starting with the first videotaped case and the first 60 s of the 5 min Pre-HL study phase, the coders examined each 1-s interval and coded the occurrence and duration of each maternal behavior described in the MBCS. Only when each of five 60 s time blocks was coded in succession for the Pre-HL session did the coders move to code the five 60 s time blocks of the Post-HL session. The same systematic steps were followed to code the two 5 min sessions for the remaining 23 maternal videotapes. Excluded from the analysis were behaviors that were not visible to coders for 20 s or more during any one 60 s time block (eg., due to the ill positioning of the video camera or when research staff walked in front of the camera) [20]. All of the 24 maternal videotapes used in the current analysis had complete data.

Of the 14,400 one-second intervals (~240 min) of coded maternal behavior (600 s for Pre-Hl + 600 s for Post-HL) (MDA group = 7000 s; control group = 7000 s) 20 % were randomly selected and subjected to inter-reliability testing, achieving k = 0.80 or better. Data were then entered into the General Sequential Querier for Windows (GSEQ 5.1). GSEQ5.1 is a unique data analysis program specially designed to calculate precise Prop-T measures of coded behavioral items by case, or by study group, or by study phase [20]. Next, we made use of GSEQ5.1 to combine Prop-T scores of coded MBCS items that were conceptually related, and that were then grouped into eight domains. Each domain was further structured into two mutually exclusive categories - the first representing typical maternal caregiving behavior and the other representing atypical behavior. Table 1 provides a summary of the eight MBCS main categories that were identified from this dataset, including definitions. Prop-T scores representing maternal atypical caregiving behavior and typical behavior for each maternal study case were then transferred to SPSS 21 to address study aims.

**Table 1:** Eight MBCS behaviors with definitions

	Definition/Description	
Coding Items	Typical maternal caregiving behavior	Atypical maternal caregiving behavior
Eye Gaze	Looking or seeking eye contact: Mother looking straight at baby’s face, or mother seeking eye contact with baby.	Looking away: Mother looking away from baby.
Engages baby and responsive to baby cues/actions.	Interactive, engages baby: Mother makes frequent attempts to interact/engage infant. Mother directly or indirectly responds to infant cues (baby crying, looking at or touching mother),	Minimal or no responsiveness to infant cues. Mother makes minimal, or no attempt to interact or to engage with the infant. Mother not responsive - may appear detached.
Embracing (positioning) of the baby	Mother embracing baby: Close, protective. Baby’s body is enclosed in mom’s embrace	No embracing or cradling. Baby laying in unsafe position. Mother does not appear to notice baby may fall off her lap.
Direction of mother’s attention	Attention directed to baby: Mother focuses attention on baby,	Attention not directed to baby. Mother seems self-absorbed, her attention is not directed to baby, to others, or to her environment.
Type and quality of facial expressions	Regular: Mother facial expression regular, smiling.	Not regular: Grimacing, crying quietly, crying excessively. Quality of facial expression is flat or mother appears disconnected or “checked out”
Comfort	Comfortable: Mother appears comfortable or she says she is comfortable.	Not comfortable: Mother appears uncomfortable (eg., mother sighs, rolls eyes) or says she is uncomfortable.
General affect	Unbothered: Mother’s generally appears unbothered sad, or worried.	Bothered: Mother appears bothered, worried, sad, upset or very upset (distraught).
Anxiety	No such display: Mother does not appear anxious	Anxious: Mother bites nails, touches hair, repetitive nervous knee movement.

MBCS definitions from Ethogram of Maternal Behavior Coding System [18]

#### Infant behavioral measures and coding procedures

The systematic procedures that Warnock et al. [19] used to continuously code each of the 21 infant pre-recorded videotapes were the same as those described above for coding the 24 maternal pre-recorded videotapes. As reported by Warnock et al. [19], the reliably established and cross-validated Neonatal Distress Pain Related Behavioral Coding Schema (ND-BSC) was used to code the infant videotapes by four coders who were trained in the ND-BSC and who were blinded to infant group. However, coders were not blinded to study phase because the laboratory technician’s hand, used to conduct the HL, was visible to the coder. The ND-BCS is an ethologically based behavioral coding schema that consists of items for coding continuously and concurrently infant motor movement, posture, respiration responsiveness and cry behavior. The procedures used to test for inter-reliability between coders, and to reduce and further analyze data in GSEQ 5.1 were also the same as those described above for coding the maternal videotaped cases. Inter-reliability between coders achieved k = 0.80 or better [19]. Sequential and comparative analysis of Prop-T scores of ND-BCS coded items led to identifying the three temporal patterns of newborn pain behavior self-regulation that were suggestive of delay in pain recovery (crying in a weak/exhausted manner, strained/erratic limb movement and immobility).

#### Data analysis

Simple descriptive statistics were obtained for all study variables and distributions of continuous variables were inspected for normality. For the total sample of 24 mothers, Mann-Whitney and Wilcoxon Signed Rank test for non-normally distributed data were used to test the hypothesis that the proportion of time that study mothers spend exhibiting atypical behavior during the 5 min Pre-HL and the 5 min Post-HL study phases would differ between MDA-exposed mothers and maternal controls. For the later, Prop-T scores of maternal behavior were analyzed when six or more mothers in either group exhibited MBCS behaviors during Pre-HL or Post-HL. Separate simple linear regressions using adjusted *R* ^2^ were performed on the total sample of 24 mothers to test whether prenatal MDA (HAM-A, HAM-D and EPDS mean scores) would predict Prop-T scores of maternal atypical caregiving behavior during the 5 min Pre-HL and 5 min Post-HL study phases. For the matched subsample of 16 mothers and their infants, we also performed simple regressions to examine if Prop-T scores of types of maternal atypical behavior during the Pre-HL phase would predict Prop-T scores of the three atypical patterns of pain behavior self-regulation in infants previously reported [19] during the HL and post-HL sub-phases of infant HL. Finally, partial correlations were used to examine the potential contribution of prenatal maternal drug use (SSRI/SU) on study outcomes as appropriate. All analyses were two-tailed with alpha set at *p* < .05, as appropriate.

## Results

### Prenatal mental health measures and maternal and infant characteristics

Of the 24 mothers, 12 had scores that exceeded the predefined cut-off for the HAM-A (range, 8–29), or the HAM-D (range, 8–29) or the EPDS (range, 11–27). These 12 mothers were allocated to the MDA-exposed group, and the 12 mothers whose scores fell below the cut-offs were allocated to the non-MDA-exposed group (maternal controls). MDA-exposed mothers had significantly higher HAM-A, HAM-D and EPDS scores than did the non MDA-exposed group of mothers (*U* = 14, *p* < .001, *r* = .75; *U* = 12, *p* < .001, *r* = .77; *U* = 11, *p* < .001, *r* = .78, respectively). Of interest, we found that ten of 12 mothers allocated to the MDA-exposed group had comorbid anxiety and depression based on exceeding cut-off scores on two or more of the tools: HAM-A + HAM-D (*n* = 5) and HAM-A + HAM-D + EPDS (*n* = 5). In the subsample of 16 mothers, eight had scores that exceeded the predefined cut-offs, and eight had scores that fell below the cut-offs. Of the eight mothers whose scores exceeded the cut-offs, six had comorbid anxiety and depression: HAM-A + HAM-D (*n* = 1) and HAM-A + HAM-D + EPDS (*n* = 5).

The 24 mothers had a mean age of 34 years (SD = 5.6) and a mean of 17.46 years of education (SD = 4.9). Most were married (63 %), Caucasian (67 %), and first-time mothers (63 %). The mode of delivery was vaginal (58.1 %) or by C-section (41.7 %). As reported in the Warnock et al. [19] study, infants had a mean gestational age of 39.75 weeks (SD = 1.53), birthweight of 3436 g (SD = .3859), and 62 % were male. There were no statistically significant group differences on maternal characteristics or on factors considered as confounders to maternal caregiving behavior such as smoking, infant gender or number of children or on infant characteristics. The preceding findings were consistent for the subsample of 16 mothers and infants.

### Association and group differences in types of caregiving behavior between mothers with and without MDA before and following infant HL

**Fig. 1:**
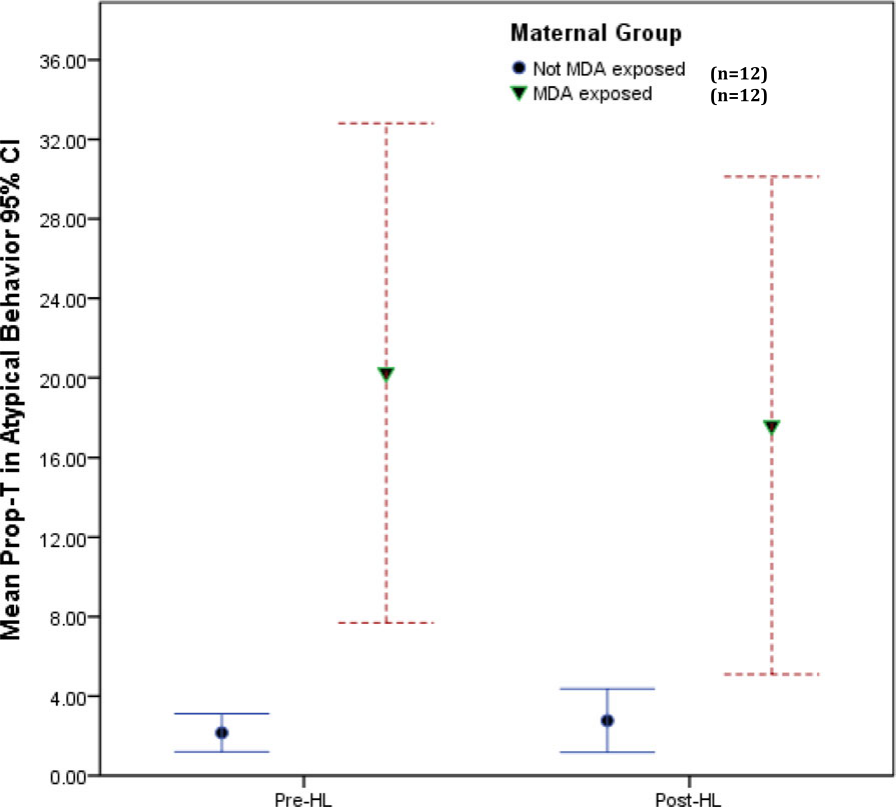
Group differences and change in Mean proportion of time (total Prop-T score, 95 % CI) that the 24 mothers in the two study groups [(MDA-exposed (*n* = 12) and Non-MDA exposed (*n* = 12)] spent exhibiting atypical caregiving behavior to infant behavioral cues while with their infant for 5 min before the infant had the HL (Pre-HL) and for 5 min after the infant had the HL (Post-HL)

Initial analysis involving the 24 mothers, showed moderate positive correlation between prenatal maternal anxiety (HAM-A) and depression (HAM-D, EPDS), and total Prop-T scores of maternal atypical caregiving behavior during routine infant HL (*r* = .604, *p* = .002; *r* = .447, *p* = .03; *r* = .631, *p* < .00, respectively). Both groups of mothers (MDA-exposed and non-MDA-exposed) exhibited typical caregiving behavior to their infants, during the 5 min Pre-HL and 5 min Post-HL study phases. However, as Fig. 1 illustrates, during the two study phases, mean scores of atypical caregiving behavior were higher for MDA-exposed mothers compared to non-MDA exposed mothers with both groups showing little change from the Pre-HL phase to the Post-HL phase. Notably, the distributions of scores were considerably more varied for the MDA-exposure mothers than they were for the non-MDA exposed mothers.

The Mann-Whitney tests were conducted to examine group differences in Prop-T spent in varying types of atypical behavior when six or more mothers in either group (MDA-exposed (*n* = 12), non-MDA exposed (*n* = 12) exhibited MBCS behaviors during the 5 min Pre-HL or the 5 min Post-H study sessions. The results revealed that during the Pre-HL phase, MDA-exposed mothers were more likely than maternal controls to not embrace (*U* = 36, *p* < .001, *r* = .55), attend (*U* = 34, *p* = .028, *r* = .45) or engage and respond to the infant’s behavioral pain cues (*U* = 36, *p* = .038, *r* = .42). Wilcoxon Signed Rank Test showed no change in Prop-T of atypical behavior between the two groups of mothers from the Pre-HL to Post-Hl study phase. However, the results showed, that although six MDA-exposed mothers spent a significant proportion of the Pre-HL phase not embracing, three of the six spent significantly less time in not embracing Post-HL (U = 40, *p* = .021, *r* = .51). Table 2 provides a summary of the preceding results. The table does not include results pertaining to atypical expression of MBCS comfort, or general affect and anxiety because the behaviors were not exhibited by enough mothers (six or more for the study phases).

**Table 2:** Proportion of time (Prop-T) study mothers spent in five MBCS atypical behavior Pre-HL and Post-HL

	Group	Phase						Z
		Pre-HL (5 min)			Post-HL (5 min)			
		n	M(SD)	Mdn	n	M(SD)	Mdn	
Looking away from baby.	Control	10	9.95 (13.89)	5.35	9	10.20 (9.49)	9.00	−314
	MDA-Exposed	12	22.67 (24.89)	15.50	12	18.72 (18.46)	12.80	−.235
Minimal or no responsiveness to baby cues/action. minimal, or no attempt to interact or to engage with the infant	Control	12	10.45 (13.70)	4.85	8	9.39 (8.86)	8.00	.000
	MDA-Exposed	12	28.72 (27.15)	20.50*	12	25.86 (38.86)	.110*	−1.34
Not embracing, cradling baby.	Control	0	.000 (.00)	.00	0	.000 (.00)	.00	.000
	MDA-Exposed	6	35.39 (45.11)	6.15*	3	9.14 (21.31)	.00	−.2.201**
Mothers attention not directed towards baby, others, or environment	Control	10	10.20 (13.88)	4.85	8	9.05 (8.56)	8.00	−.639
	MDA-Exposed	12	32.25 (33.30)	20.50*	10	20.77 (23.75)	12.65	.523
Type and quality of facial expression not regular.	Control	10	17.00 (28.29)	4.20	6	7.19 (14.96)	.50	−1.27
	MDA-Exposed	9	29.28 (36.26)	20.50	9	30.52 (36.65)	19.85	−.533

Values represent mean proportion of time (number of seconds behavior expressed/total number of seconds of the observation session) based on 24 mothers (12 in MDA-exposed grp, 12 in Control group), *n* total number of mothers in each group who exhibited the behavior (minimum of 6 required in either phase). *p < .05 Mann Whitney *U* test; **p < .05 Wilcoxon signed-rank test

### Relationships between prenatal maternal mental health measures and maternal atypical caregiving behavior before and after infant HL

Separate simple linear regressions involving the 24 mothers were performed to determine if prenatal MDA (mean scores of HAM-D, or HAM-A or EPDS) would predict Prop-T scores of maternal atypical caregiving behavior during the Pre-HL and Post-HL study phases. Results showed HAM-D scores predicted Prop-T scores of atypical caregiving behavior during the Pre-HL (β = .46, *t* = 2.46, *p* = .022), but more so during the Post-HL phase (β = .66, *t* = 4.16, *p* = .01). HAM-A and EPDS scores also predicted Prop-T scores of maternal atypical caregiving behavior, but only during Post-HL: HAM-A (*β* = .64, *t* = 3.95, *p* < .001); EPDS (*β* = .64, *t* = 3.92, *p* < .001).

HAM-D scores accounted for a smaller proportion of variance in atypical behavior during Pre-HL [adjusted *R* ^2^ = .180, *F* (1, 22) = 6.05, *p* = .022] than during Post-HL [adjusted *R* ^2^ = .416, *F* (1, 22) = 17.35, *p* = .01]. HAM-A and EPDS scores also accounted for up to 38 % of the variance in atypical expression Post-HL: HAM-A [adjusted *R* ^2^ = .389, *F* (1, 22) = 15.66, *p* < .001], EPDS [adjusted *R* ^2^ = .385, *F* (1, 22) = 15.39, *p* < .001].

### Relationship between maternal atypical caregiving behavior during Pre-HL and infant pain self-regulation behavior during the sub-phases of infant HL (HL and post-HL)

Results of the final simple linear regressions that involved the matched subsample of 16 mothers and their infants showed the Prop-T that mothers spent in not engaging/ responding or attending to infant’s behavioral cues during the 5 min Pre-HL phase, predicted the Prop-T that infant offspring spent exhibiting strained/erratic limb movements (β = .552, *t* = 2.47, *p* = .027) and immobility (β = .566, *t* = 2.57, *p* = .022) during the actual HL. Prop-T scores of these maternal behavior explained 25 % of the variance in infant strained/erratic limb movement [adjusted *R* ^2^ = .255, *F* (1, 14) = 6.14, *p* = .027] and 27 % in immobility [adjusted *R* ^2^ = .272, *F* (1, 14) = 6.60, *p* = .022] during the actual HL.

We also found the Prop-T the 16 mothers spent not engaging or attending during the 5 min Pre-HL phase, predicted the Prop-T their infants spent in weak/strained cry during the post-HL sub-phase (β = .690, t = 3.56, *p* = .003, and β = .518, *t* = 2.26, *p* = .040, respectively). The Prop-T the mothers spent in not engaging or attending Pre-HL explained up to 21 % of the variance in infant weak/strained cry post-HL subphase: [adjusted *R* ^2^ = .216, *F* (1, 14) = 5.132, *p* = .040].

## Discussion

The overall goal of this basic observation study using time based analytic approaches was to clarify the significance of MDA and its potential underlying impact on maternal caregiving behavior and infant pain behavior self-regulation, in the first few days following infant birth. Acknowledging the complex and many multiple pathways of association possible, the precise and in-depth time-based findings generated in this study contribute robust foundational measures of maternal and infant behavior that could not have been generated using conventional approaches. Main findings suggest that MDA may underlie atypical maternal caregiving behavior and that maternal caregiving behavior may mediate infant pain behavior self-regulation.

As hypothesized, MDA-exposure was associated with type and temporal quality of atypical maternal caregiving behavior before and after infant HL. During the Pre-HL phase, MDA-exposed mothers were more likely than maternal controls to not engage /respond or attend to infant behavioral cues Pre-HL–with little change Post-HL. That three of the six MDA-exposed mothers spent less time in not embracing their infant Post-HL suggest change toward more responsive caregiving behavior. But given the very small number of mothers who exhibited change, further study is required before conclusion can be drawn. The considerable variability in Prop-T that the 12 mothers with MDA spent exhibiting atypical behavior may be because 83 % of these mothers had cut-off scores indicating moderate to severe comorbidity.

Our findings of association between MDA and time-based measures of maternal caregiving before and after routine infant HL are consistent with findings of a lack of responsiveness and insensitivity behavior in mothers with postpartum depression and/ or anxiety reported in studies that did not involve infant pain [31, 32]. They are also consistent with time-based findings of the Warnock et al. [18] study that showed a lack of engagement and responsiveness in mothers with MDA as they held their infants during the 2 min HL. Our findings extend those findings, in that our group of MDA-exposed mothers exhibited lack of engagement and responsive to infant cues while with their infants, 5 min before the 2 min HL and 5 min after the HL. Taken together, findings of the prior study [18] and this study suggest that MDA-exposed mothers may express atypical patterning in caregiving behavior over the course of infant HL.

In our study, factors that have been acknowledged to influence maternal atypical caregiving behavior such as the number of children, gender of an infant or maternal smoking did not present as confounders. However, one factor that we did not examine and that may have contributed to our findings of maternal atypical caregiving behavior is a lack of sleep or physical exhaustion in mothers, especially given that we sampled mothers within days of giving birth. In a recent study, mothers with an EPDS score of >12 at one week postpartum were significantly more likely to report being tired [33]. In addition, maternal fatigue in that study was strongly associated with new onset of depressive symptoms. These maternal variables may have interactive or confounding effects and therefore warrant consideration in future studies.

Our second and third hypotheses that MDA exposure (as measured by 2nd trimester HAM-D, or HAM-A or EPDS scores) would predict Prop-T scores of maternal atypical caregiving behavior and that maternal atypical caregiving behavior Pre-HL would be associated with the three patterns of atypical infant pain behavior self-regulation reported previously [19] were both supported. Similar to the findings reported by Warnock et al. [18], we found that prenatal depression and anxiety was positively associated with atypical expression of caregiving behavior. Our findings extend the prior findings [18] in that prenatal depression, and anxiety (based on independent analysis of HAM-A, HAM-D and /or EPDS scores) accounted for up to 41 % of the variance in atypical expression of caregiving behavior Post-HL. Controlling for prenatal maternal drug use (SSRIs, crack cocaine) had little impact on these findings. Our approach to examining the relationship of MDA to Prop-T scores of maternal caregiving contributed knowledge on the distinct contributions MDA during the 2nd trimester based on independent analysis of HAM-A, or HAM-D or EPDS scores. Future studies with large sample sizes will help determine which of the three measures best predict atypical maternal caregiving and the contributions of comorbidity and pregnancy anxiety at each trimester.

We also found that the Prop-T that the 16 mothers in the subsample spent in not embracing and in not engaging/responding or attending to infant behavioral cues during the 5 min Pre-HL phase were associated with the Prop-T their infants spent in immobility and in strained/erratic limb movement during actual HL, and in weak/strained cry during the post- HL sub-phase. These findings extend the findings by Warnock et al. [19] in that we included time-based measures of maternal caregiving in the concurrent analysis. While the prior findings [19] suggest that infant ability to self-regulate to and from painful stimuli may be impacted by prenatal exposure to MDA, the current findings suggest that type/quality of maternal caregiving behavior may play a mediatory role in this dynamic. However, these results were based on a sample of16 mothers and their infants. As such, the results are preliminary, but they warrant further study. If the maternal and infant findings are again observed in future studies, convergent findings may provide the basis for the development of early clinical interventions that promote the caregiving behavior and stress regulatory role of mothers with MDA. Newborn ability to behaviorally regulate to stressors is a marker of neurodevelopment [2], and early indication of sustained delay in self-regulation in the full-term necessitate early assessment and treatment.

The dearth of similar studies in the context of neonatal pain makes it difficult to compare our findings. Other studies that did not involve infant pain have reported linkages between prenatal maternal depression and/or anxiety and postnatal alteration in maternal caregiving behavior and later child outcomes. For example, findings have shown that prenatal anxiety predicts depressive symptoms during the postpartum [34] and that it accounts for 10–15 % of adverse behavioral, emotional, cognitive and mental health outcomes in later childhood [10, 35] such as low social competencies and adaptive functioning [36]. In contrast, other findings have shown that maternal insensitivity, but not antenatal psychiatric diagnosis, predicts infant responsiveness in a free play situation at four months post-partum [16]. Potential reasons for the discrepant findings between that study and our findings is that we examined caregiving behavior of mothers with MDA during the newborn period and because the findings were based on the use of ethologically driven methods that are specifically designed to help explain biological mechanisms underlying observed behavior.

The in-depth descriptions of the types and temporal qualities of maternal caregiving behavior that were elucidated in this study are clinically relevant. Clinicians with interests in mother driven interventions for neonatal pain may use the findings to consider inclusion of mothers with MDA and their infants. These mothers may represent the 36 % of women who decline participation [8], and it is unclear if these dyads would benefit from the regulatory properties of the interventions. There is, however, emerging evidence that maternal-infant skin-to-skin contact intervention for neonatal pain may have regulatory benefits for both mothers and infants regardless of maternal mental health status [37]. Clinical recruitment and future research of this kind will help support mothers with MDA and their infants and as noted, may provide the basis for potential modification of the interventions, as appropriate.

This study has limitations that necessitate cautious interpretation of study findings. Although this basic observation study yielded foundational data based on a huge amount of time-based measures, the small subject sample size limits the generalizability of the findings. Some of the study mothers had taken antidepressant medications and/or drugs (SSRIs, crack cocaine) during pregnancy. Although we controlled for these effects statistically, these mothers may exhibit a unique behavioral profile due to impacts of the drugs and chronic exposures to environmental adversity and stress. It was beyond the present study to have distinguished any behavioral differences, and this may be an area for future dedicated research. Findings were based on independent measures of prenatal depression and anxiety and the study focused on maternal and infant behavior. Targeting comorbidity at recruitment, making use of multivariate analysis and obtaining physiological and other measures (eg., measures of maternal and infant cortisol levels, heart rate variability) in future larger sized samples will help clarify pathways of association.

## Conclusions

Sensitive and responsive maternal caregiving behavior strengthens infant self-regulatory capacities, but this powerful regulatory role may be diminished in some women with MDA. Our findings suggest that mothers and infants with 2nd trimester MDA exposure exhibit atypical behavior during a routine infant pain event and that MDA may underlie these atypical patterning in behavior. The findings are consistent with findings of studies that have examined associations between MDA, maternal caregiving behavior that did and did not involve infant pain, and that were conducted during the postpartum. We recommend early inclusion of mothers with MDA and their infants in interventions aimed to strengthen the early mother-infant interaction and a mother’s caregiving regulatory role. MDA and maternal caregiving behavior must be considered in future infant pain studies and to examine potential confounding effects of MDA on effectiveness of mother driven interventions for neonatal pain. We highlight the importance of future research on maternal mental health throughout the trajectory of the perinatal period, particularly during the early newborn period, and on development and implementation of early strength-based interventions.

## Abbreviations

MDA: Second-trimester prenatal maternal exposure to depression and/ or anxiety
HL: Infant heel lance pain procedure
Prop-T: The proportion of time that an individual or group spends in a particular behavior during an observation session(s)
NAS: Neonatal abstinence syndrome
SSRIs: Selective Serotonin Re-uptake Inhibitor antidepressant medication
HPA: Hypothalamic–pituitary-adrenal axis
